# RACIAL AND ETHNIC DISPARITIES IN THE EFFECTS OF COVID-19 ON EMPLOYMENT DISRUPTION AND FINANCIAL PRECARITY

**DOI:** 10.1093/geroni/igad104.0890

**Published:** 2023-12-21

**Authors:** Dawn Carr, Amanda Sonnega, Rebekah Carpenter, Katy (Qiuchang) Cao, Qize Chen

**Affiliations:** Florida State University, Tallahassee, Florida, United States; University of Michigan, Ann Arbor, Michigan, United States; Florida State University, Tallahassee, Florida, United States; Florida State University, Tallahassee, Florida, United States; University of Michigan, Ann Arbor, Michigan, United States

## Abstract

The COVID-19 pandemic disproportionately influenced minoritized individuals, with significantly higher rates of infection and death. One reason for this higher rate is hypothesized to relate to occupational environments of older minoritized populations had going into the pandemic, which also make them more likely to experience disruption in employment due to factors such as illness, and consequently, more financial consequences of the pandemic than their white counterparts. The current project evaluates race and ethnicity differences in disruptions in employment among older workers during the pandemic, and the extent to which those disruptions were related to the inability to pay monthly bills such as rent/mortgage, utilities, medical bills, food, or debt-related payments. Using data drawn from the Health and Retirement Study, including a COVID-19 survey collected in September 2021, this study evaluates older workers who were employed prior to the start of the pandemic to evaluate differences by race-ethnicity in whether and for what reasons they experienced job disruptions, and the extent to which these disruptions were associated with an inability to pay monthly bills. We further evaluate the extent to which being unable to pay monthly bills is moderated by pre-pandemic financial vulnerabilities. Results indicate that Black and Hispanic older people experience more significant financial consequences of job disruptions, and that pre-pandemic financial vulnerabilities significantly moderate the relationship between race-ethnicity and COVID-related financial consequences of job disruptions. To conclude, the policy-implications of these results will be discussed, particularly potential factors that may decrease financial vulnerability during retirement among minoritized populations.

